# Draft genomic sequence of a selenite-reducing bacterium, *Paenirhodobacter enshiensis* DW2-9^T^

**DOI:** 10.1186/s40793-015-0026-9

**Published:** 2015-07-18

**Authors:** Dan Wang, Fengqiu Zhu, Xiaoli Zhu, Shixue Zheng, Rui Wang, Gejiao Wang

**Affiliations:** State Key Laboratory of Agricultural Microbiology, College of Life Sciences and Technology, Huazhong Agricultural University, Wuhan, 430070 PR China; Tobacco Company of Enshi, Hubei Province, Enshi, 445000 Hubei PR China

**Keywords:** *Rhodobacteraceae*, *Paenirhodobacter enshiensis*, Selenite-reducing bacterium, Genome sequence, Comparative genomics

## Abstract

*Paenirhodobacter enshiensis* is a non-photosynthetic species that belongs to family *Rhodobacteraceae*. Here we report the draft genome sequence of *Paenirhodobacter enshiensis* DW2-9^T^ and comparison results to the available related genomes. The strain has a 3.4 Mbp genome sequence with G + C content of 66.82 % and 2781 protein-coding genes. It lacks photosynthetic gene clusters and putative proteins necessary in Embden-Meyerhof-Parnas (EMP) pathway, but contains proteins in Entner-Doudoroff (ED) pathway instead. It shares 699 common genes with nine related *Rhodobacteraceae* genomes, and possesses 315 specific genes.

**Electronic supplementary material:**

The online version of this article (doi:10.1186/s40793-015-0026-9) contains supplementary material, which is available to authorized users.

## Introduction

Family *Rhodobacteraceae* belongs to *Proteobacteria* which was established by Garrity *et al*. [1] and contains 105 genera including both chemoorganotrophic and photoheterotrophic bacteria. The type genus was *Rhodobacter* which was first proposed by Imhoff *et al*. in 1984 [2] and comprised of only photosynthetic species [3–8]. In 2013, we proposed *Paenirhodobacter enshiensis* DW2-9^T^ to represent one of the non-photosynthetic genera of *Rhodobacteraceae* [9]. The main differences between *Paenirhodobacter* and its closest relative *Rhodobacter* are their photosynthetic characteristics and major polar lipid types [9]. *Haematobacter* is another non-photosynthetic genus of *Rhodobacteraceae* [10] and the main difference between *Haematobacter* and *Paenirhodobacter* is the cultivation condition [9–11].

So far, the genus *Paenirhodobacter* contains only one species, *Paenirhodobacter enshiensis*. The main characters of *P. enshiensis* DW2-9^T^ are non-photosynthetic and possessing phosphatidylglycerol, phosphatidylethanolamine and aminophospholipid as the major polar lipids [9]*.* In addition, we found that strain *P. enshiensis* DW2-9^T^ was able to reduce soluble selenite (Se^4+^) into insoluble elemental selenium nanoparticle (Se^0^). Since Se^0^ is less bioavailable, this strain could potentially been used in bioremediation of soil or water with selenite-contamination.

In order to provide genomic information for elucidating the mechanism of bacterial selenite reduction, as well as the taxonomic study, we performed genome sequencing of strain *P. enshiensis* DW2-9^T^, together with its close relatives *Haematobacter missouriensis* CCUG 52307^T^ [10] and *Haematobacter massiliensis* CCUG 47968^T^ [11]. In this study, we report the genomic features of *P. enshiensis* DW2-9^T^ and the comparison results to the close relatives. This microorganism is not belonged to a larger genomic survey project.

## Organism information

### Classification and features

Strain *P. enshiensis* DW2-9^T^ was isolated from soil near a sewage outlet of the Bafeng pharmaceutical factory, Enshi city, Hubei province, PR China. The general features of *P. enshiensis* DW2-9^T^ are shown in Table 1. The 16S rRNA gene based phylogenetic tree showing the phylogenetic relationships of *P. enshiensis* DW2-9^T^ to other taxonomically classified type strains of the family *Rhodobacteraceae* could be found in our previous study [9].

**Table 1:** Classification and general features of *P. enshiensis* DW2-9^T^ [12]

MIGS ID	Property	Term	Evidence code^a^
	Classification	Domain *Bacteria*	TAS [13]
		Phylum *Proteobacteria*	TAS [14]
		Class *Alphaproteobacteria*	TAS [15]
		Order *Rhodobacterales*	TAS [16]
		Family *Rhodobacteraceae*	TAS [1, 17]
		Genus *Paenirhodobacter*	TAS [9]
		Species *Paenirhodobacter enshiensis*	TAS [9]
		Type strain DW2-9^T^ (Accession #JN797511)	
	Gram stain	negative	TAS [9]
	Cell shape	rod	TAS [9]
	Motility	non-motile	TAS [9]
	Sporulation	non-sporulating	NAS
	Temperature range	4-42 °C	TAS [9]
	Optimum temperature	28 °C	TAS [9]
	pH range; Optimum	5–8; 7	TAS [9]
	Carbon source	aetate, propionate, pyruvate, fumarate, malate, citrate, succinate, D-glucose, D-fructose, D-xylose and maltose	TAS [9]
MIGS-6	Habitat	soil	TAS [9]
MIGS-6.3	Salinity	0- 3 % NaCl (w/v), optimal at 0 %	TAS [9]
MIGS-22	Oxygen requirement	facultatively anaerobic	TAS [9]
MIGS-15	Biotic relationship	free-living	TAS [9]
MIGS-14	Pathogenicity	non-pathogen	NAS
MIGS-4	Geographic location	Enshi city, Hubei province, P. R. China	TAS [9]
MIGS-5	Sample collection	2010	TAS [9]
MIGS-4.1	Latitude	29°52′55′′ N	TAS [9]
MIGS-4.2	Longitude	110°03′21′′ E	
MIGS-4.4	Altitude	not reported	

^a^Evidence codes - IDA: Inferred from Direct Assay; TAS: Traceable Author Statement (i.e., a direct report exists in the literature); NAS: Non-traceable Author Statement (i.e., not directly observed for the living, isolated sample, but based on a generally accepted property for the species, or anecdotal evidence). These evidence codes are from the Gene Ontology project [18]

**Fig. 1:**
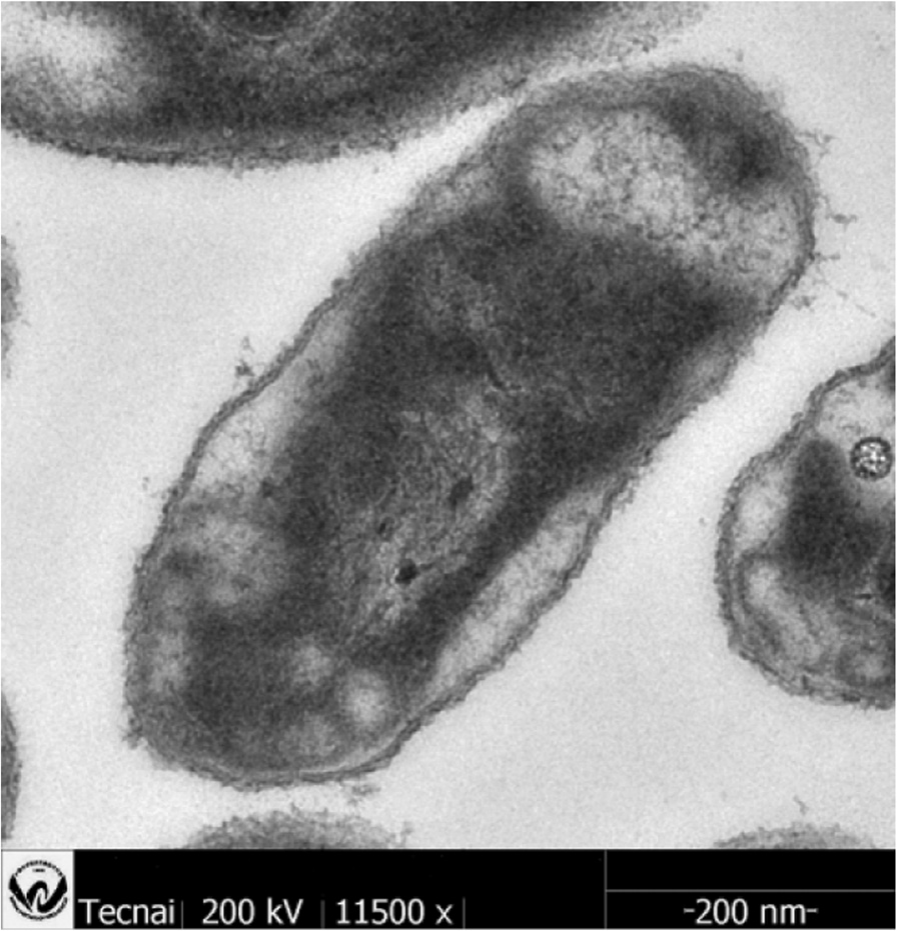
A TEM image of ultrathin sections for *P. enshiensis* DW2-9^T^ cells. The scale bar represents 200 nm

Strain DW2-9^T^ is Gram-negative, facultatively anaerobic, non-motile, non-photosynthetic, and rod-shaped (Fig. 1). Cells are 0.9-1.2 μm long and 0.3-0.6 μm wide. Colonies are convex, circular, smooth and white after 2 days of incubation on modified Biebl & Pfennig’s agar at 30 °C [9]. The strain was able to reduce 0.2 mmol/L of sodium selenite (Na_2_SeO_3_) into Se^0^ within 2 days when grown in Luria-Bertani medium.

The chemotaxonomic features include phosphatidylglycerol, phosphatidylethanolamine and aminophospholipid as the major polar lipids, ubiquinone-10 as the major quinone and C_16:0_, C_18:1_*ω*7*c*, C_19:0_ cyclo *ω*8*c* and summed feature 3 (one or more of iso-C_15:0_ 2-OH, C_16:1_*ω*6*c* and C_16:1_*ω*7*c*) as the major cellular fatty acids of [9].

## Genome sequencing information

### Genome project history

Strain *P. enshiensis* DW2-9^T^ was sequenced by Majorbio Bio-pharm Technology Co., Ltd, Shanghai, China. The draft genome sequence of strain *P. enshiensis* DW2-9^T^ has been deposited at DDBJ/EMBL/GenBank under accession number JFZB00000000. The version described in this study is the first version JFZB01000000 and consists of sequences JFZB01000001-JFZB01000112. The project information are summarized in Table 2

**Table 2:** Project information

MIGS ID	Property	Term
MIGS-31	Finishing quality	High-quality draft
MIGS-28	Libraries used	Illumina Paired-End library (300 bp insert size)
MIGS-29	Sequencing platforms	Illumina Miseq 2000
MIGS-31.2	Fold coverage	222 ×
MIGS-30	Assemblers	SOAPdenovo v1.05
MIGS-32	Gene calling method	GeneMarkS^+^
	Locus TAG	CG50
	Genbank ID	JFZB00000000
	Genbank Date of Release	August 17, 2014
	GOLD ID	Gi0077179
	Bioproject	PRJNA239787
MIGS-13	Source material identifier	DW2-9^T^
	Project relevance	Genome comparison

.

### Growth conditions and genomic DNA preparation

Strain *P. enshiensis* DW2-9^T^ was grown aerobically in LB medium at 28°C for 36 h. The DNA was extracted, concentrated and purified using the QiAamp kit according to the manufacturer’s instruction (Qiagen, Germany).

### Genome sequencing and assembly

The genome of *P. enshiensis* DW2-9^T^ was sequenced by Illumina technology [19]. An Illumina standard shotgun library was constructed and sequenced using the Illumina MiSeq 2000 platform, which generated 3,128,974 reads totaling 941.8 Mbp.

All original sequence data can be found at the NCBI Sequence Read Archive [20]. The following steps were performed for removing low quality reads: (1) removed the adapter in the reads, (2) cut the 5’ end bases which were not A, T, G, C, (3) filtered the reads which have a quality score lower than 20, (4) filtered the reads which contained N more than 10 percent, (5) removed the reads which have the length less than 25 bp after processed by the previous four steps. The processed reads were assembled by SOAPdenovo v1.05 [21].

The final draft assembly contained 153 contigs in 85 scaffolds. The total size of the genome is 3.4 Mbp and the final assembly is based on 764.6 Mbp of Illumina data, which provides an average 222× coverage of the genome. The simulated genome of *P. enshiensis* DW2-9^T^ is a set of contigs ordered against the complete genome of *Rhodobacter capsulatus* SB1003 (NC_013034) using Mauve software [22].

### Genome annotation

The draft genome of *P. enshiensis* DW2-9^T^ was annotated through the RAST server version 2.0 [23] and the National Center for Biotechnology Information Prokaryotic Genome Annotation Pipeline, which combines the gene caller GeneMarkS^+^ [18] with the similarity-based gene detection approach.

Protein function classification was performed by WebMGA [24] with E-value cutoff 1-e^10^. The transmembrane helices were predicted by TMHMM Server v. 2.0 [25]. Internal gene clustering was performed by OrthoMCL using Match cutoff of 50 % and E-value Exponent cutoff of 1-e^5^ [26, 27]. Signal peptides in the genome were predicted by SignalP 3.0 server [28]. The translation predicted CDSs were also used to search against the Pfam protein family database [29], KEGG [30] and the NCBI Conserved Domain Database through the Batch web CD-Search tool [31].

## Genome properties

**Fig. 2:**
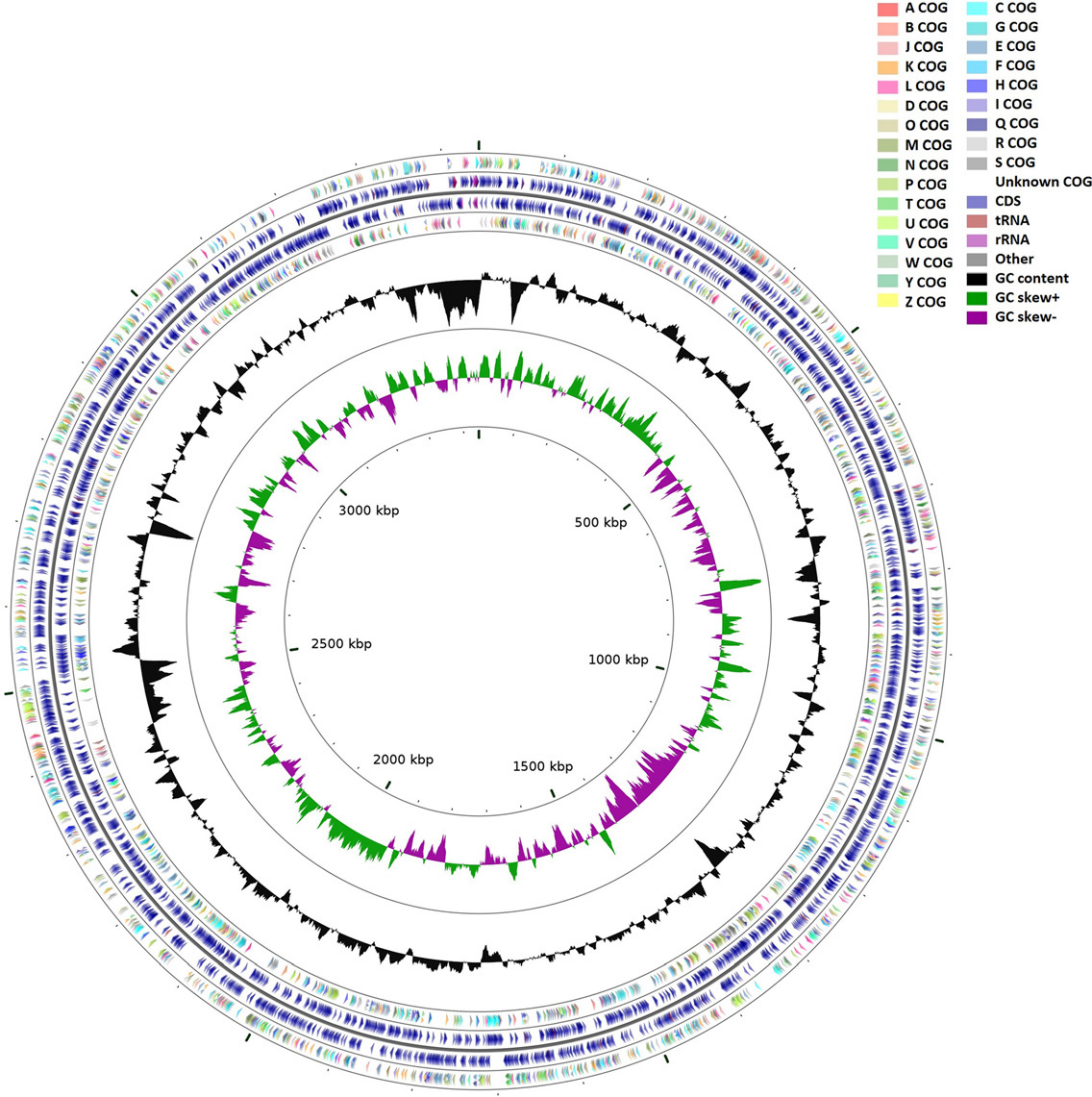
A graphical circular map of the genome performed with CGview comparison tool [32]. From outside to center, ring 1, 4 show protein-coding genes colored by COG categories on forward/reverse strand; ring 2, 3 denote genes on forward/reverse strand; ring 5 shows G + C% content plot, and the innermost ring shows GC skew

The whole genome of P. enshiensis DW2-9^T^ is 3,439,591 bp in length, with an average GC content of 66.82 %, and is distributed in 112 contigs (>200 bp). The genome properties and statistics are summarized in Table 3 and Fig. 2. A total of 2781 protein-coding genes are identified and 78.99 % of them are distributed into COG functional categories (Table 4).

**Table 3:** Nucleotide content and gene count levels of the genome

Attribute	Genome (total)	
	Value	% of total^a^
Genome size (bp)	3,439,591	100
DNA coding (bp)	2,662,806	77.41
DNA G + C (bp)	2,298,404	66.82
Total genes^b^	2856	
RNA genes	56	
Pseudo genes	19	
Protein-coding genes	2781	100
Genes in internal clusters	1156	41.57
Genes with function prediction	2061	74.11
Genes assigned to COGs	2196	78.99
Genes with Pfam domains	2495	89.74
Genes with signal peptides	717	25.79
Genes with transmembrane helices	588	21.15
CRISPR repeats	3	

^a^The total is based on either the size of the genome in base pairs or the total number of protein coding genes in the annotated genome

^b^Also includes 19 pseudogenes, 10 RNA genes, 45 rRNAs and 1 ncRNA

**Fig. 3:**
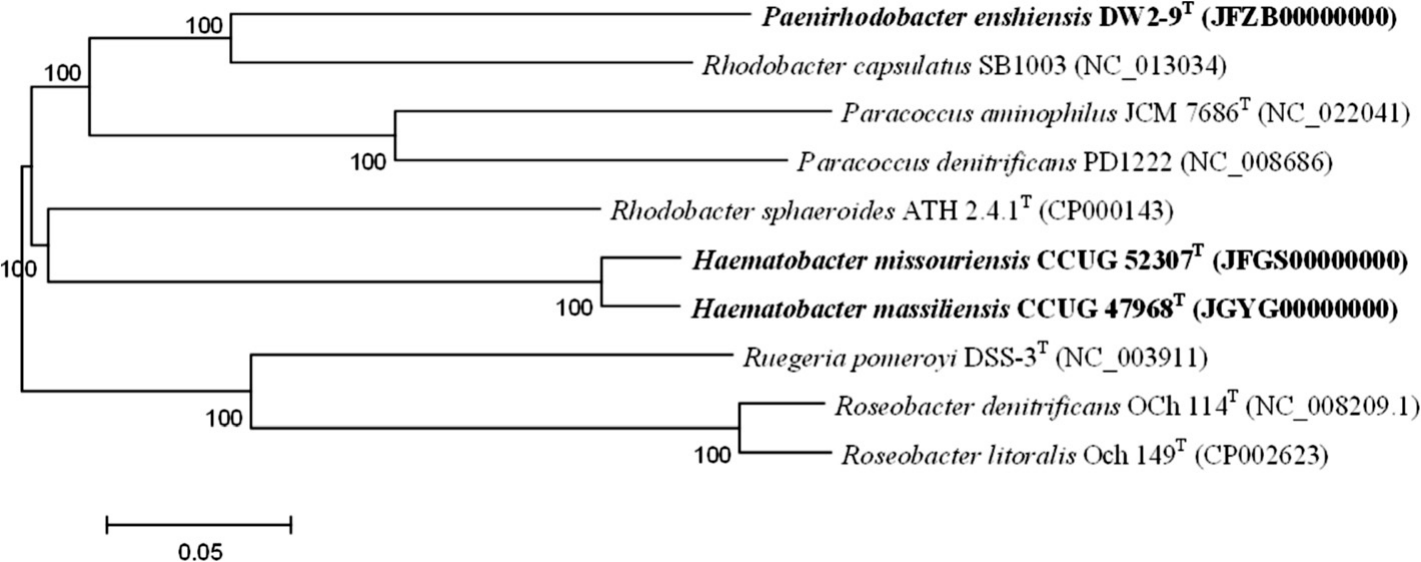
A phylogenetic tree highlighting the phylogenetic position of *P. enshiensis* DW2-9^T^. The conserved protein was analyzed by OrthoMCL with Match Cutoff 50 % and E-value Exponent Cutoff 1-e^5^ [26, 27]. The phylogenetic tree was constructed based on the 699 single-copy conserved proteins shared among the ten genomes. The phylogenies were inferred by MEGA 5.05 with NJ algorithm [38], and 1000 bootstrap repetitions were computed to estimate the reliability of the trees. The genome accession numbers of the strains are shown in parenthesis

**Fig. 4:**
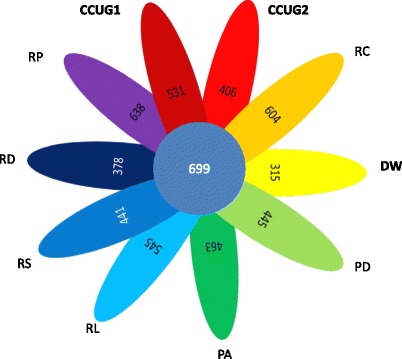
Ortholog analysis of *P. enshiensis* DW2-9^T^ and nine *Rhodobacteraceae* genomes conducted using OrthoMCL with Match cutoff of 50 % and E-value Exponent cutoff of 1-e^5^. The total numbers of shared proteins of the ten genomes were tabulated and presented as a Venn diagram. Abbreviations for strain names: DW, *P. enshiensis* DW2-9^T^; CCUG1, *Haematobacter missouriensis* CCUG 52307^T^; CCUG2, *Haematobacter massiliensis* CCUG 47968^T^; RC, *Rhodobacter capsulatus* SB1003; RS, *Rhodobacter sphaeroides* ATH 2.4.1^T^; PA, *Paracoccus aminophilus* JCM 7686^T^; PD, *Paracoccus denitrificans* PD1222^T^; RD, *Roseobacter denitrificans* OCh 114; RL, *Roseobacter litoralis* Och 149^T^; RP, *Ruegeria pomeroyi* DSS-3^T^

**Fig. 5:**
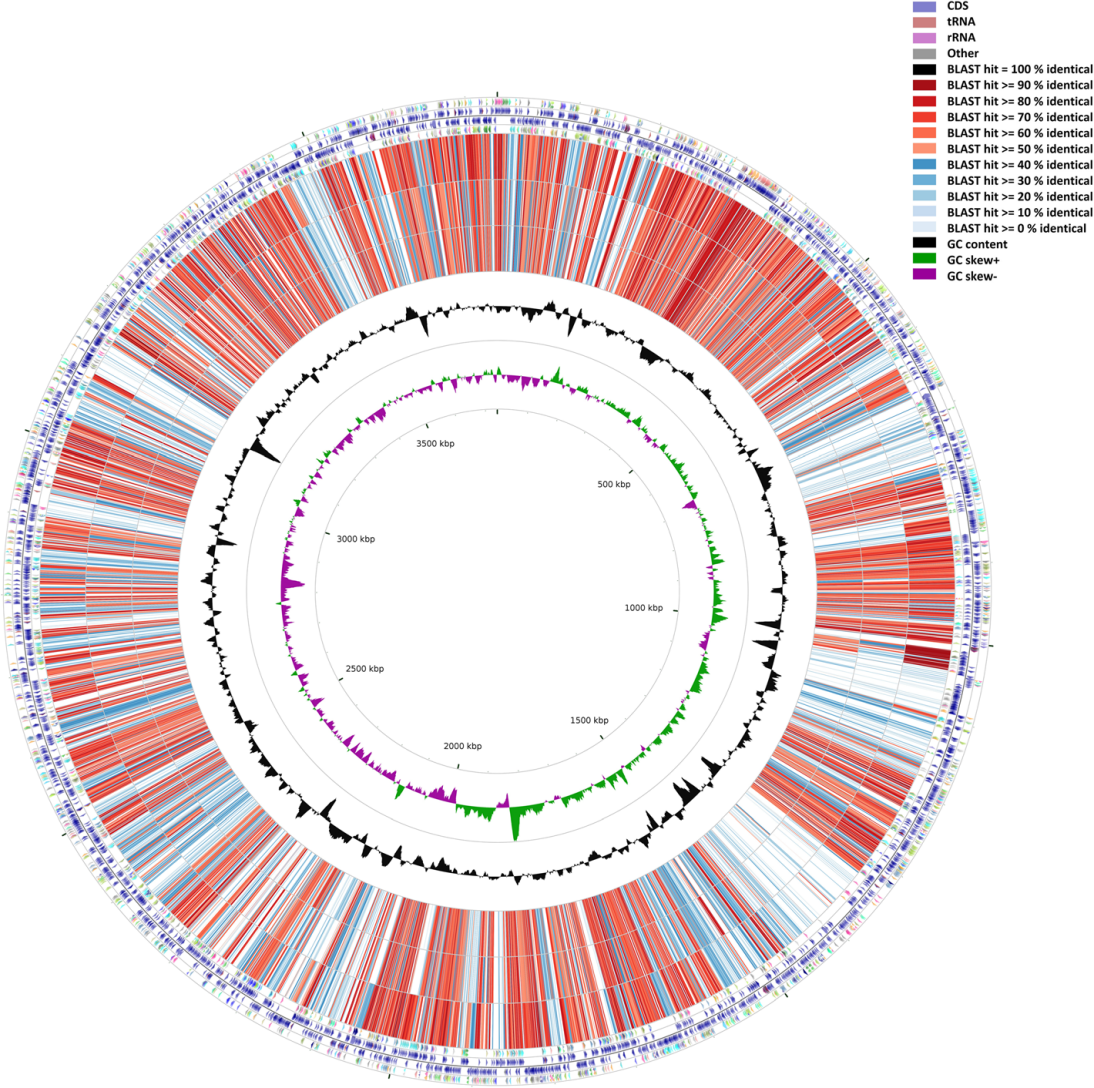
A graphical circular map of the comparison between reference strain *Rhodobacter capsulatus* SB 1003 and the three strains sequenced in this study. From outside to center, rings 1, 4 show protein-coding genes colored by COG categories on forward/reverse strand; rings 2, 3 denote genes on forward/reverse strand; rings 5, 6, 7 show the CDS vs CDS BLAST results of *Rhodobacter capsulatus* SB 1003 with *P. enshiensis* DW2-9^T^, *H. massiliensis* CCUG 47968^T^ and *H. missouriensis* CCUG 52307^T^, respectively; ring 8 shows G + C% content plot, and the innermost ring shows GC skew

**Table 4:** Number of genes associated with the 25 general COG functional categories

Code	Value	% of total^a^	Description
J	154	5.54	Translation
A	0	0.00	RNA processing and modification
K	137	4.93	Transcription
L	93	3.34	Replication, recombination and repair
B	1	0.04	Chromatin structure and dynamics
D	25	0.89	Cell cycle control, mitosis and meiosis
Y	0	0.00	Nuclear structure
V	36	1.29	Defense mechanisms
T	83	2.98	Signal transduction mechanisms
M	124	4.46	Cell wall/membrane biogenesis
N	29	1.04	Cell motility
Z	0	0.00	Cytoskeleton
W	0	0.00	Extracellular structures
U	53	1.91	Intracellular trafficking and secretion
O	92	3.31	Posttranslational modification, protein turnover, chaperones
C	155	5.57	Energy production and conversion
G	97	3.49	Carbohydrate transport and metabolism
E	385	13.84	Amino acid transport and metabolism
F	78	2.80	Nucleotide transport and metabolism
H	116	4.17	Coenzyme transport and metabolism
I	84	3.02	Lipid transport and metabolism
P	162	5.83	Inorganic ion transport and metabolism
Q	51	1.83	Secondary metabolites biosynthesis, transport and catabolism
R	263	9.46	General function prediction only
S	186	6.69	Function unknown
-	585	21.01	Not in COGs

^a^The total is based on the total number of protein coding genes in the annotated genome

## Insights from the genome sequence

### Profiles of metabolic network and pathway

Strain DW2-9^T^ is facultatively anaerobic and can utilize a variety of sole carbon substrates, including acetate, propionate, pyruvate, fumarate, malate, citrate, succinate, D-glucose, D-fructose and maltose [9]. Genome analysis showed that this strain has the corresponding enzymes to utilize these sole carbon sources and to catabolize them via different pathways (mainly by the TCA cycle and pentose phosphate). Especially in glycolysis, strain *P. enshiensis* DW2-9^T^ lacks the key enzyme 6-phosphofructokinase that is essential in Embden-Meyerhof-Parnas (EMP) pathway. Instead, it contains 6-phosphogluconate dehydratase (KFI24690) and 2-keto-3-deoxyphosphogluconate aldolase (KFI24689) that were characterized in Entner-Doudoroff (ED) pathway. 

All key genes necessary for fatty acid biosynthesis are present. All genes required for *de novo* synthesis of 15 common amino acids are present. Genes for biosynthesis of Ala, Asn, Met, Tyr and His are not present. 

As a non-photosynthetic bacterium, the known photosynthetic gene clusters, including the *bch* genes, *puf* genes and *crt* genes were not found in the genome of *P. enshiensis* DW2-9^T^.

In this study, strain DW2-9^T^ was found to be capable of reducing selenite into selenium nanoparticle. It has been reported that low-molecular weight thiols such as glutathione [33] and cysteine [34], nitrite reductase [35], fumarate reductase [36], glutathione reductase and thioredoxin reductase [37] could reduce selenite into elemental selenium. In the genome of strain DW2-9^T^, all the encoding genes of the respective enzymes mentioned above were found (e.g. KFI26491, KFI30857, KFI28250, KFI28810, KFI29698, KFI24274 and KFI29723).

### Comparisons with other *Rhodobacteraceae* genomes

The genomic sequence of strain DW2-9^T^ was compared to nine available *Rhodobacteraceae* strains (*Haematobacter missouriensis* CCUG 52307^T^, *Haematobacter massiliensis* CCUG 47968^T^, *Rhodobacter capsulatus* SB1003, *Rhodobacter sphaeroides* ATH 2.4.1^T^, *Paracoccus aminophilus* JCM 7686^T^, *Paracoccus denitrificans* PD1222, *Ruegeria pomeroyi* DSS-3^T^, *Roseobacter denitrificans* OCh 114^T^ and *Roseobacter litoralis* Och 149^T^). OrthoMCL was used again to perform ortholog clustering analysis with Match cutoff of 50% and E-value Exponent cutoff of 1-e^5^ [26, 27]. A total of 699 shared protein sequences were obtained and a neighbor-jointing (NJ) phylogenomic tree [38] was constructed (Fig. 3). The phylogenomic result based on the 699 proteins is generally consistent with the 16S rRNA gene tree [9]. The ortholog clustering analysis also revealed that strain *P. enshiensis* DW2-9^T^ has 315 strain-specific genes, which potentially contributes to genus-specific features distinguishing *Paenirhodobacter* from other genera (Fig. 4).

In this study, we also sequenced the genomes of two members of *Haematobacter* genus, strain *H. missouriensis* CCUG 52307^T^ [10] and *H. massiliensis* CCUG 47968^T^ [11]. The draft genome sequences were 3.9 and 4.1 Mbp, the G+C contents were 64.31 % and 64.56 %, and the numbers of predicted protein-coding genes were 3,612 and 3,806, respectively. Figure 5 shows the genome comparison results of strain *P. enshiensis* DW2-9^T^, *H. missouriensis* CCUG 52307^T^ and *H. massiliensis* CCUG 47968^T^ using CGview comparison tool [32]. Table 5 presents the difference of the gene number (in percentage) in each COG category between strain *P. enshiensis* DW2-9^T^, *H. missouriensis* CCUG 52307^T^ and *H. massiliensis* CCUG 47968^T^.

**Table 5:** Percentage of genes associated with the 25 general COG functional categories for *P. enshiensis* DW2-9^T^, *H. missouriensis* CCUG 52307^T^ and *H. massiliensis* CCUG 47968^T^

Code	COG description	*P. enshiensis* DW2-9^T^	*H. missouriensis* CCUG 52307^T^	*H. massiliensis* CCUG 47968^T^
J	Translation	5.54	4.26	4.23
A	RNA processing and modification	0.00	0.00	0.00
K	Transcription	4.93	4.82	4.99
L	Replication, recombination and repair	3.34	3.27	3.28
B	Chromatin structure and dynamics	0.04	0.03	0.00
D	Cell cycle control, mitosis and meiosis	0.89	0.97	0.92
Y	Nuclear structure	0.00	0.00	0.00
V	Defense mechanisms	1.29	1.11	0.89
T	Signal transduction mechanisms	2.98	2.19	2.57
M	Cell wall/membrane biogenesis	4.46	3.71	3.76
N	Cell motility	1.04	0.69	0.58
Z	Cytoskeleton	0.00	0.00	0.00
W	Extracellular structures	0.00	0.00	0.00
U	Intracellular trafficking and secretion	1.91	2.05	1.45
O	Posttranslational modification, protein turnover, chaperones	3.31	3.16	3.10
C	Energy production and conversion	5.57	5.20	5.10
G	Carbohydrate transport and metabolism	3.49	3.82	3.60
E	Amino acid transport and metabolism	13.84	10.96	11.09
F	Nucleotide transport and metabolism	2.80	2.25	2.29
H	Coenzyme transport and metabolism	4.17	3.63	3.47
I	Lipid transport and metabolism	3.02	4.43	4.60
P	Inorganic ion transport and metabolism	5.83	6.17	7.12
Q	Secondary metabolites biosynthesis, transport and catabolism	1.83	2.71	2.52
R	General function prediction only	9.46	9.63	9.77
S	Function unknown	6.69	6.84	7.02
-	Not in COGs	21.01	18.11	17.66

## Conclusions

Genomic analysis of *P. enshiensis* DW2-9^T^ revealed a high degree of consistency between genotypes and phenotypes, especially in sole carbon source utilization and non-photosynthetic nature. Genome sequencing of strain *P. enshiensis* DW2-9^T^ provides extra supports for its taxonomic classification. The genome sequence of strain DW2-9^T^ also provides insights to better understand the molecular mechanisms of selenite reduction. In addition, this strain could potentially been used for bioremediation of environmental selenite-contamination.

The associated MIGS records are shown in Additional file 1: Table S1.

## Additional file

Additional file 1: Table S1. Associated MIGS record.

## Abbreviations

RAST: Rapid annotation using subsystem technology
KEGG: Kyoto encyclopedia of genes and genomes
