# Supplementary material for: Draft genomic sequence of a selenite-reducing bacterium, Paenirhodobacter enshiensis DW2-9T
Source: Stand Genomic Sci. 2015 Jul 18;10:38. doi: 10.1186/s40793-015-0026-9 (PMC4517658; doi:10.1186/s40793-015-0026-9)
Supplement: Additional file 1: Table S1. — Associated MIGS record. [file 40793_2015_26_MOESM1_ESM.docx]

**Associated MIGS Record**

**Table S1.** Associated MIGS record

| **MIGS-ID** | field name | description |
| --- | --- | --- |
| **MIGS-1** | Submit to INSDC/Trace archives |  |
| **1.1** | PID | JFZB00000000.1 |
| **1.2** | Trace Archive |  |
| **MIGS-2** | MIGS CHECK LIST TYPE | bacteria |
| **MIGS-3** | Project Name | The National Natural Science Foundation of  China (31470227) |
| **MIGS-4** | Geographic Location |  |
| **4.1** | Latitude | 29°52′55′′ N |
| **4.2** | Longitude | 110°03′21′′ E |
| **4.3** | Depth | not reported |
| **4.4** | Altitude | not reported |
| **MIGS-5** | Time of Sample collection | 2010 |
| **MIGS-6** | Habitat (EnvO) |  |
| **6.1** | temperature | not reported |
| **6.2** | pH | not reported |
| **6.3** | salinity | not reported |
| **6.4** | chlorophyll | not reported |
| **6.5** | conductivity | not reported |
|  |  |  |
| **6.6** | light intensity | not reported |
| **6.7** | dissolved organic carbon (DOC) | not reported |
| **6.8** | current | not reported |
| **6.9** | atmospheric data | not reported |
| **6.10** | density | not reported |
| **6.11** | alkalinity | not reported |
| **6.12** | dissolved oxygen | not reported |
| **6.13** | particulate organic carbon (POC) | not reported |
| **6.14** | phosphate | not reported |
| **6.15** | nitrate | not reported |
| **6.16** | sulfates | not reported |
| **6.17** | sulfides | not reported |
| **6.18** | primary production | not reported |
| **MIGS-7** | Subspecific genetic lineage | not reported |
| **MIGS-9** | Number of replicons | 1 |
| **MIGS-10** | Extrachromosomal elements | unknown |
| **MIGS-11** | Estimated Size | 3.4 Mbp |
| **MIGS-12** | Reference for biomaterial or Genome report | not reported |
| **MIGS-13** | Source material identifiers | KCTC 15169^T^, CCTCC AB2011145^T^ |
| **MIGS-14** | Known Pathogenicity | non-phytopathogenic |
|  |  |  |
| **MIGS-15** | Biotic Relationship | fee-living |
| **MIGS-16** | Specific Host | not reported |
| **MIGS-17** | Host specificity or range (taxid) | not reported |
| **MIGS-18** | Health status of Host | not reported |
| **MIGS-19** | Trophic Level | not reported |
| **MIGS-22** | Relationship to Oxygen | facultatively anaerobic |
| **MIGS-23** | Isolation and Growth conditions | doi: 10.1099/ijs.0.050351-0 |
| **MIGS-27** | Nucleic acid preparation | extracted, concentrated and  purified using the QiAamp kit |
| **MIGS-28** | Library construction |  |
| **28.1** | Library size | 300 bp |
| **28.2** | Number of reads | 3,128,974 |
| **28.3** | vector | 0 |
| **MIGS-29** | Sequencing method | Illumina MiSeq 2000 |
| **MIGS-30** | Assembly |  |
| **30.1** | Assembly method | SOAPdenovo v1.05 |
| **30.2** | estimated error rate | 1 error within 1,000 bases |
| **30.3** | method of calculation | estimation from  quality scores |
| **MIGS-31** | Finishing strategy |  |
| **31.1** | Status | High-quality draft |
| **31.2** | coverage | 222 × |
| **31.3** | contigs | 153 |
| **MIGS-32** | Relevant SOPs | not reported |
| **MIGS-33** | Relevant e-resources | not reported |
